# Mapping of quantitative trait loci related to primary rice root growth as a response to inoculation with *Azospirillum* sp. strain B510

**DOI:** 10.1080/19420889.2018.1502586

**Published:** 2018-08-04

**Authors:** Sachiko Masuda, Kazuhiro Sasaki, Yuri Kazama, Chiharu Kisara, Shoko Takeda, Eiko Hanzawa, Kiwamu Minamisawa, Tadashi Sato

**Affiliations:** a Graduate School of Life Sciences, Tohoku University, Sendai, Japan; b RIKEN, Center for Sustainable Resource Science, Yokohama City, Japan; c Institute for Sustainable Agro-ecosystem Services, Graduate School of Agricultural and Life Sciences, The University of Tokyo, Tokyo, Japan; d Graduate School of Agricultural Science, Tohoku University, Sendai, Japan

**Keywords:** Primary rice root growth, quantitative trait locus, *Azospirillum* sp. strain B510, plant growth-promoting bacteria, plant genotype

## Abstract

*Azospirillum* sp. strain B510 has been known as the plant growth-promoting endophyte; however, the growth-promotion effect is dependent on the plant genotype. Here, we aimed to identify quantitative trait loci (QTL) related to primary root length in rice at the seedling stage as a response to inoculation with B510. The primary root length of “Nipponbare” was significantly reduced by inoculation with B510, whereas that of “Kasalath” was not affected. Thus, we examined 98 backcrossed inbred lines and four chromosome segment substitution lines (CSSL) derived from a cross between Nipponbare and Kasalath. The primary root length was measured as a response to inoculation with B510, and the relative root length (RRL) was calculated based on the response to non-inoculation. Three QTL alleles, *qRLI-6* and *qRLC-6* on Chromosome (Chr.) 6 and *qRRL-7* on Chr. 7 derived from Kasalath increased primary root length with inoculation (RLI), without inoculation, (RLC) and RRL and explained 20.2%, 21.3%, and 11.9% of the phenotypic variation, respectively. CSSL33, in which substitution occurred in the vicinity region of *qRRL-7*, showed a completely different response to inoculation with B510 compared with Nipponbare. Therefore, we suggest that *qRRL-7* might strongly control root growth in response to inoculation with *Azospirillum* sp. strain B510.

## Introduction

Bacterial endophytes ubiquitously colonize the internal tissues of plants worldwide [1]. The beneficial effects of endophytes on host plants, such as increased crop yield and protection from pathogens, have been well characterized [1,2]. The genus *Azospirillum* [3] promotes plant growth and is thus used as a plant-promoting agronomic application [4]. *Azospirillum* sp. strain B510 was isolated from the surface-sterilized stem of a rice plant (*Oryza sativa* L.) [5]. This bacterium can increase the disease resistance of the host plant [6] and promote the growth of newly generated leaf and shoot biomass in rice plants under greenhouse conditions [7]. Under field conditions, *Azospirillum* sp. B510 increases tiller numbers in rice, resulting in grain yield increases of 17% [7]. However, inoculation with *Azospirillum* sp. B510 can reduce rice plant growth depending on plant genotype and environmental conditions; inoculation with *Azospirillum* sp. B510 significantly increased the tiller number in most indica cultivars, whereas tiller numbers in several japonica cultivars were significantly decreased under standard nitrogen fertilization [8]. These results suggested that yield enhancements could be link to the combinations of strains and host plant genotypes as well as nitrogen fertilization level. Indeed, inoculation of 12 mustard (*Brassica juncea* L.) genotypes with a mixture of *Azospirillum* strains showed biomass changes of – 30% to 160%, compared with the control [9]. The differential growth yields depended on strain-host genotype combinations were also observed in maize, wheat, and rice [10–12]. They are likely controlled via strong interactions between the plant and *Azospirillum*. However, the molecular mechanisms or genetic loci associated with responses to inoculation with *Azospirillum* strains in different rice cultivars have not yet been identified.

Colonization studies suggested that endophytes use the natural cracks at the lateral roots and root hairs emergence site as entry sites in rhizosphere [13–15]. Thus, plant-endophyte interaction actively occurs in the rhizosphere. Successful inoculation results in changes the root morphology and phytohormonal balance which are also attributed to successful inoculation [16]. *A. brasilense* Sp245 inhibits root growth of wheat, whereas the root length is recovered as similar length to control when a mutant strain lacking indole acetic acid (IAA) productivity is inoculated [17]. These results suggest that the effect on root growth is due to IAA produced by *A. brasilense* Sp245. *Azospirillum* inoculation changes the proﬁles of rice secondary metabolites [18] and the transcriptional level of genes related to auxin and ethylene signaling depend on the combination of bacterial strain and plant genotype [16]. Therefore, root morphology and secondary metabolite proﬁling such as plant hormones revealed specific responses of host plant to inoculation with *Azospirillum* strains.

In the present study, we clarified that inoculation of Nipponbare, japonica cultivar, and Kasalath, indica cultivar, with B510 resulted in differences in primary root growth. In Nipponbare, inoculation with B510 showed a negative effect; primary root growth was delayed compared to plants which were not inoculated. In contrast, when Kasalath was inoculated, primary root growth showed little or no change compared to plants without inoculation. To identify quantitative trait loci (QTL) related to the primary root growth in rice we used 98 backcrossed inbred lines (BILs) and four chromosome segment substitution lines (CSSL9, CSSL31, CSSL32, and CSSL33) derived from a cross between Nipponbare and Kasalath. We evaluated primary root growth as an index of response to inoculation with B510. Our results might generate new insights into the interaction of *Azospirillum* sp. with the host plant.

## Materials and methods

### Plant materials

A total of 98 BILs and four CSSLs derived from a cross between the *indica* rice cultivar Kasalath and the *japonica* rice cultivar Nipponbare were used. These lines were developed by the Rice Genome Project of the National Institute of Agrobiological Sciences, Japan and kindly provided along with information on their genotypic profiles by the Rice Genome Resource Centre (http://www.rgrc.dna.affrc.go.jp/). BILs, CSSLs, and the parental cultivars were grown and harvested in paddy fields of the Experimental Farm Station, Graduate School of Life Sciences, Tohoku University, Kashimadai, Osaki, Miyagi, Japan.

### Bacterial inoculation and plant growth condition

*Azospirillum* sp. B510 was cultured in nutrient broth (Difco Laboratories, Detroit, MI, USA) at 30°C for 16 h, centrifuged at 5,000 rpm for 3 min, washed twice with sterile distilled water, and a bacterial suspension was prepared in sterile water. The bacterial suspension was diluted to 1.3 × 10^7^–1.3 × 10^2^ cells ml^−1^ to examine the dose response of B510 on the parental cultivars, Nipponbare and Kasalath. A concentration range of 1.6–3.6 × 10^6^ cells ml^−1^ was used for the inoculation of BILs and CSSLs. Rice seeds were germinated on two filter papers (Toyo Roshi Kaisha Ltd., Tokyo, Japan) in a Petri dish (6 cm in diameter). The filter papers and petri dishes were autoclaved at 121°C for 30 min before use. The seeds by immersion in water at 55°C for 20 min and then, in 2.4% sodium hypochlorite (Wako Pure Chemical Industries, Osaka, Japan) for 15 min. Finally, the seeds were rinsed over 5 times and shaken 3 times for 5 min each with sterile water. Ten seeds were placed on filter papers, inoculated with 5 ml of the bacterial suspension at different concentrations if needed, and incubated at 30°C for 2 d. We treated the seeds with 5 ml of sterile water as a control (without inoculation). A rice seed with or without B510 inoculation was placed on a 0.25% agarose gel (Wako Pure Chemical Industries) with 1/10 strength hydroponic solution [19] in a glass tube and incubated at 26°C for 6 d in a growth chamber (light 16h, dark 8h). Each inoculation experiment was performed with 5 plants. After incubation, the primary root length was measured, and the relative root length was calculated as follows:

Relative root length (RRL) (%) = average of root length with inoculation (RLI)/average of root length without inoculation (RLC) × 100.

### Genotyping and QTL analysis

BILs were genotyped using 245 genome-wide restriction fragment length polymorphisms (RFLPs) as described by Lin et al. [20]. Linkage analysis was performed using Mapmaker/EXP 3.0 [21], whereas QTL analysis using QTL Cartographer 2.5 [22]. For composite interval mapping, the log-likelihood (LOD) thresholds (2.9 for root length with inoculation and 3.1 for root length with no inoculation and relative root length) were selected from 1,000 permutations tested at the 5% level. The percentage of total phenotypic variation explained by QTL was estimated for each trait and expressed by the *R^2^* value. Two QTL positions on the same chromosome were considered different when the distance of the nearest marker was greater than 10 cM. To confirm the effect of *qRRL-7*, RLC and RLI were compared in CSSLs substituted with Kasalath segments in the vicinity of region *qRRL-7* in a Nipponbare genetic background. Significant differences between RLC and RLI in each CSSL and parental line were calculated using *t*-tests (*n *= 10).

## Results

**Figure 1.**
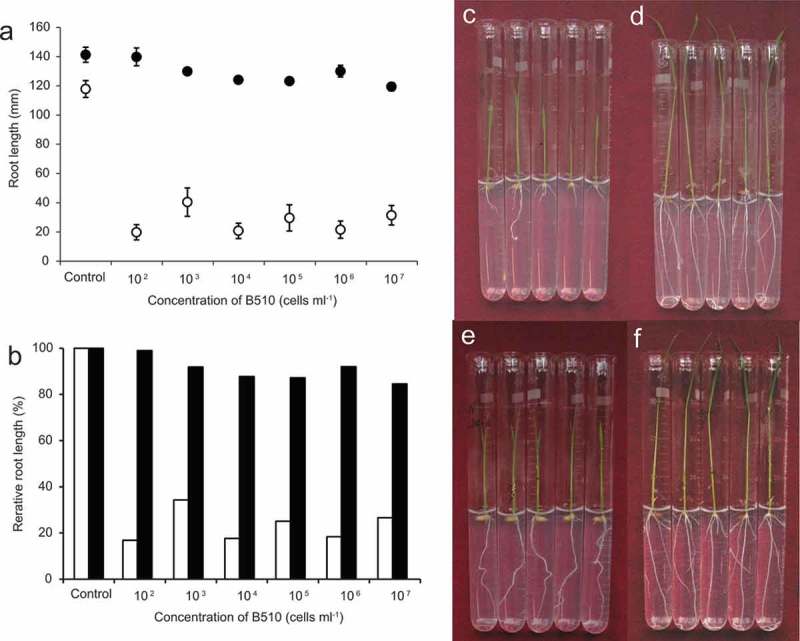
Root length and relative root length of “Nipponbare” and “Kasalath” seedlings in response to inoculation with *Azospirillum* sp. strain B510 (10^2^ cells ml^−1^) compared with the control (without inoculation) at 6 days post-inoculation. (a) Root length of Nipponbare (open circle) and Kasalath (closed circle) seedlings (*n* = 5); (b) Relative root length of Nipponbare (open bar) and Kasalath (closed bar) seedlings. Nipponbare (c and e) and Kasalath (d and f) seedlings with (c and d) or without (e and f) inoculation.

The primary root growth of Nipponbare was significantly reduced in response to inoculation with *Azospirillum* sp. B510 (Figure 1). The primary root growth of Nipponbare in response to inoculation with 10^2^ cells ml^−1^ was lower than 40 mm, whereas that without inoculation was 118 mm (Figure 1(a,c,e)). In contrast, the primary root growth of Kasalath was slightly decreased with the increasing *Azospirillum* sp. B510 concentration, but no significant differences were observed (Figure 1(a,d,f). To identify the gene trait loci of primary root growth in response to *Azospirillum* sp. B510 inoculation, we subsequently inoculated to BILs and CSSLs with this bacterium at the concentration of 10^6^ cells ml^−1^. Length of primary root growth is used as an index of QTL analysis.

**Figure 2.**
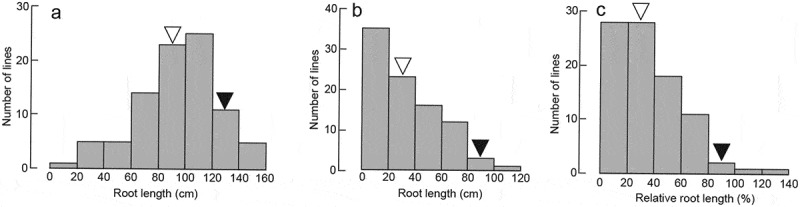
Frequency distribution of (a) root length in response to without inoculation; (b) root length in response to inoculation with *Azospirillum* sp. strain B510; and (c) relative root length of 98 backcrossed inbred lines derived from a cross between “Kasalath” (black arrowhead) and “Nipponbare” (white arrowhead).

**Figure 3.**
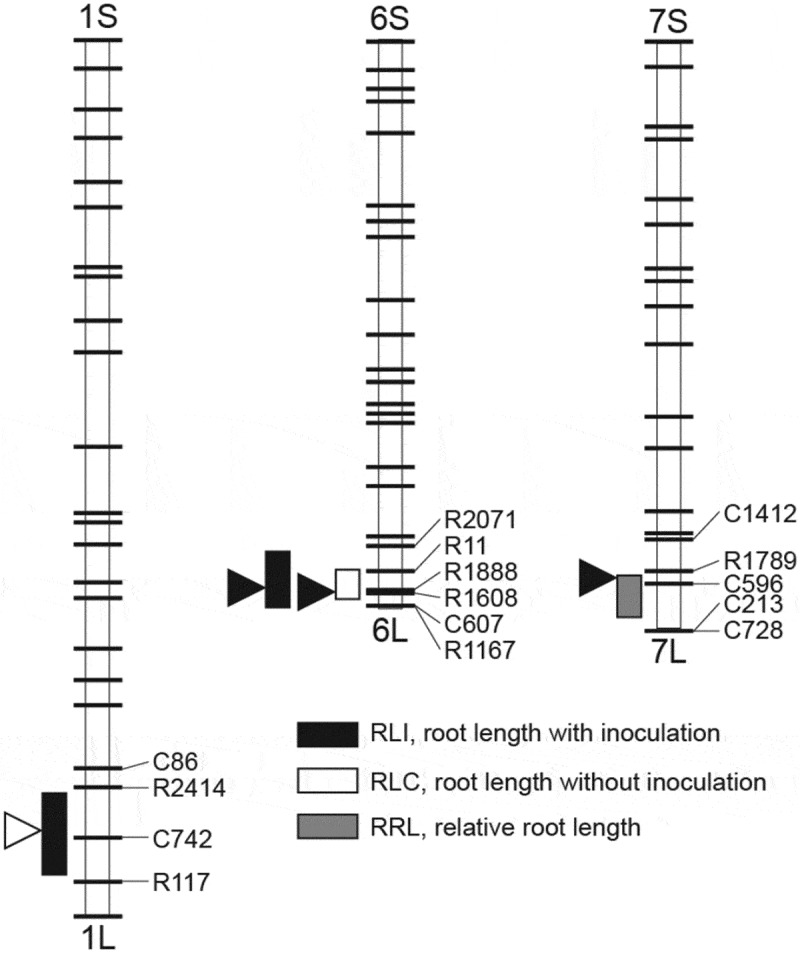
Mapping of quantitative trait loci (QTL) for root length in response to without inoculation (RLC) or inoculation with *Azospirillum* sp. strain B510 (RLI), and relative root length (RRL) on a rice linkage map. Black, white, and grey boxes indicate the QTL region for RLI, RLC, and RRL, respectively. Box length indicates the 1-LOD confidence interval of putative QTL. White and black arrowheads show the positions of LOD peaks that indicate positive alleles from “Nipponbare” and “Kasalath,” respectively.

The frequency distribution of RLI, RLC, and RRL showed continuous variation (Figure 2). Based on this phenotypic variation, QTL analyzes were performed, and two putative QTL associated with RLI, one QTL associated with RLC, and one QTL associated with RRL were detected (Figure 3, Table 1). The putative QTL for RLI were mapped near C724 on Chromosome (Chr.) 1 (*qRLI-1*) and near R1888 on Chr. 6 (*qRLI-6*); for RLC near R1608 on Chr. 6 (*qRLC-6*); and for RRL near R1789 on Chr. 7 (*qRRL-7*) (Table 1). The alleles *qRLI-6, qRLC-6*, and *qRRL-7* derived from Kasalath increased RLI, RLC, and RRL and explained 20.2%, 21.3%, and 11.9% of the phenotypic variation, respectively (Table 1). The allele *qRLI-1* derived from Nipponbare increased RLI and explained 9.7% of the phenotypic variation (Table 1).

**Table 1.** Location and effect of quantitative trait loci (QTLs) related to the root length in response to inoculation or no inoculation with *Azospirillum* sp. strain B510 and the relative root length.

Trait ^a^	QTL	Chr.	Interval ^b^	LOD	*R^2^* ^c^ (%)	*A* ^d^
RLI	*qRLI-1*	1	*R2414/C724*	3.4	9.7	19.6
	*qRLI-6*	6	*R11/R1888*	6.2	20.2	−25.1
RLC	*qRLC-6*	6	*R1888/R1608*	6.2	21.3	−31.1
RRL	*qRRL-7*	7	*R1789/C596*	3.7	11.9	−29.3

^a^ RLI, root length in response to inoculation; RLC, root length in response to no inoculation, RRL, relative root length

^b^ The nearest RFLP marker to the QTL is underlined.

^c^ Proportion of the phenotypic variation explained by the nearest marker of QTL.

^d^ Additive effect of the allele from Nipponbare compared with that from Kasalath.

* Putative QTLs with significant LOD scores on 1,000 permutations tested at the 5% level.

**Figure 4.**
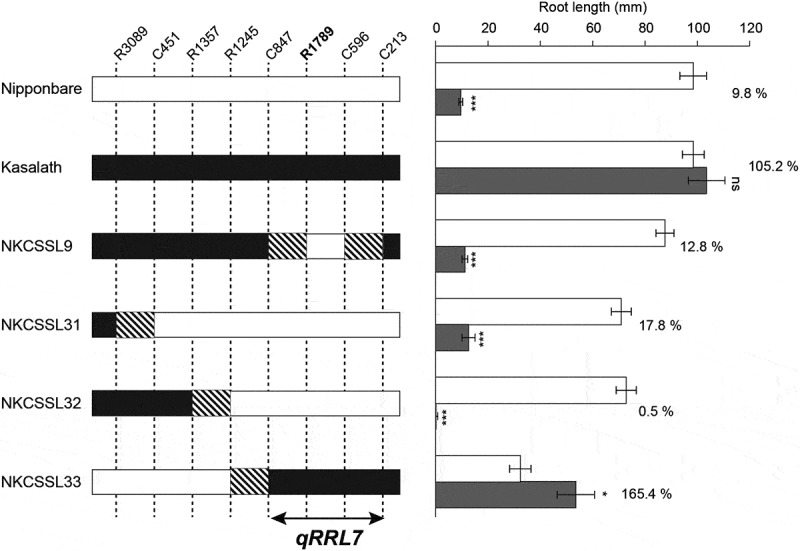
Graphical representation of the root length of “Nipponbare,” “Kasalath,” and chromosome segment substitution lines (CSSLs) in response to no inoculation (white) or inoculation with *Azospirillum* sp. strain B510 (grey). Percentages indicate the relative root length of each line. * and *** indicate significant differences between no inoculation and inoculation at *P* < 0.05 and *P* < 0.001, respectively.

To confirm the effect of *qRRL-7*, we compared the primary root growth of Nipponbare, Kasalath, CSSL9, CSSL31, CSSL32, and CSSL33. CSSL9, CSSL31, and CSSL32 showed a significantly lower RLI than RLC, whereas RRL ranged from 0.5% to 12.8% with no significant differences compared with that of Nipponbare. In contrast, the RRL of CSSL33 was 165.4% and significantly different from that of Nipponbare and other CSSLs. Based on the phenotypic and genotypic data of CSSLs, we mapped the location of *qRRL-7* between C847 and C213 (Figure 4).

## Discussion

In this study, the primary root growth of Nipponbare and Kasalath responded to inoculation with *Azospirillum* sp. B510. The bacterium caused a stronger reduction in root length in Nipponbare at much lower inoculum concentrations (10^2^ cells ml^−1^, Figure 1) but not in Kasalath. The B510 triggers the induction of the genes potentially involved in hormone signaling such as auxin, salicylic acid and ethylene in rice roots, the transcriptional changes of those genes, however, is dependent on rice cultivar [16]. The expression of the genes such as auxin and ethylene signaling genes are downregulated when B510 is inoculated to Nipponbare. These results suggested that root growth inhibition of Nipponbare by B510 could depend on the plant hormones.

We succeeded to map QTL related to primary rice root growth in response to inoculation with *Azospirillum* sp. B510. On the long arm of Chr. 6, we detected a QTL for RLI (*qRLI-6*) as well as a QTL for RLC (*qRLC-6*) based on the phenotypic variation of BILs in the primary root growth in response to with or without B510 inoculation, respectively. These results indicate that *qRLC-6* controls the primary root growth at the early seedling stage and probably affects *qRLI-6* in this study. In this region, a major QTL, namely *qRL6.1*, associated with root length has been previously detected in a population derived from a cross between the *japonica* cultivar Koshihikari and Kasalath [23]. The allele *qRL6.1* derived from Kasalath increased the root length in response to various nitrogen concentrations under hydroponic conditions [23]. The allele *qRLC-6* might be identical to *qRL6.1*, since both QTLs are derived from Kasalath and increased the root growth under the different condition such as hydroponic and glass tube conditions. We also detected a QTL for RRL (*qRRL-7*) that explained the variation in the primary root growth between Nipponbare and Kasalath. Therefore, *qRRL-7* might strongly control root growth in response to inoculation with *Azospirillum* sp. B510 and explain related differences between Nipponbare and Kasalath. The allelic effect of *qRRL-7* was also confirmed based on the phenotype of CSSL33 (Table 1, Figure 4). CSSL33, which substituted the vicinity region of *qRRL-7*, showed completely different response to inoculation with B510 compared with Nipponbare. Based on the phenotypic and genotypic data of CSSLs, we succeeded to map the location of *qRRL-7* between C847 and C213 (Figure 4). In the candidate genomic region of *qRRL-7*, the expression of *Os07g0664000* is induced by B510 inoculation in *japonica* cultivars [16]. *Os07g0664000* probably encodes short-chain dehydrogenases/reductases, which are enzymes of the NAD(P)(H)-dependent oxidoreductases family [24]. In the amino acid sequences of *Os07g0664000* in Nipponbare and Kasalath, two amino acid substitutions (R55G and R113H) were found between them (Figure S1). These results suggested that those amino acid residues may be involved in the different primary root growth by B510 inoculation. In addition, *Os07g0664000* expressed as well by infection with the fungus phytopathogen *Magnaporthe oryzae* [25]. Therefore, *qRRL-7* might be also involved in the interaction of microorganisms, including bacteria and fungi, with the host plant. However, further research is needed to validate whether *Os07g0664000* is the responsible gene of *qRRL-7* and also reveal the underlying molecular mechanism that controls the root growth in response to *Azospirillum* sp. B510 inoculation on Nipponbare and Kasalath. To this end, using inoculated CSSL33 with *Azospirillum* sp. B510 and evaluating agronomic traits, including the tiller number, under paddy field conditions might help to understand the function of *qRRL-7*.

Overall, inoculation with B510 increased the tiller number of a *japonica* rice cultivar, resulting in increased grain yield under paddy field conditions [7]. However, the positive effect on the tiller number has been found to depend on rice genotype [8]. Thus, expanding the practical usage of bio-fertilizers, including *Azospirillum* sp. B510, requires an understanding of the mechanisms of interaction between rice genotypes and inoculants.
