# Supplementary material for: Mapping of quantitative trait loci related to primary rice root growth as a response to inoculation with Azospirillum sp. strain B510
Source: Commun Integr Biol. 2018 Aug 4;11(3):1–6. doi: 10.1080/19420889.2018.1502586 (PMC6132424; doi:10.1080/19420889.2018.1502586)
Supplement: Supplemental Material [file kcib-11-03-1502586-s001.pdf]

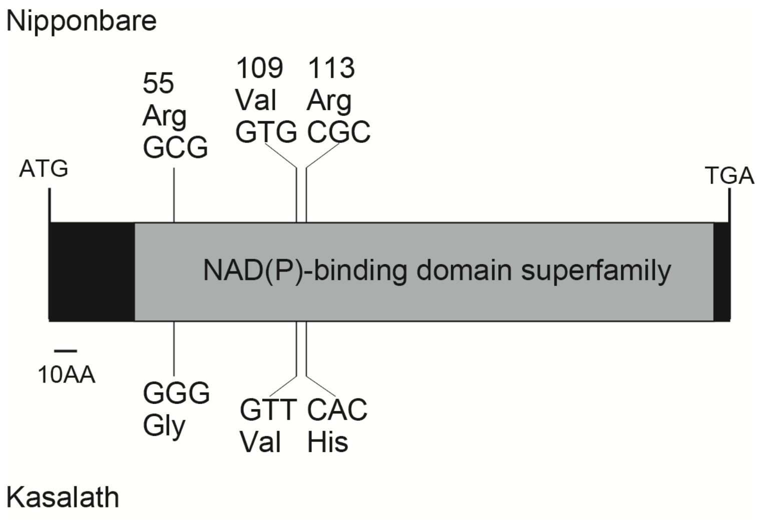

Fig. S1 Comparing the amino acid sequences of Os07g0664000 in Nipponbare and Kasalath. Upper and lower polymorphism of genome and amino acid sequence are from Nipponbare and Kasalath. Order number shows on Nipponbare amino acid sequence.
